# Response to Commentary: Efficacy and Safety of Transcranial Direct Current Stimulation as an Add-on Treatment for Bipolar Depression: A Randomized Clinical Trial

**DOI:** 10.3389/fnhum.2019.00218

**Published:** 2019-07-02

**Authors:** Andre R. Brunoni, Bernardo Sampaio-Junior

**Affiliations:** ^1^Department of Internal Medicine, Faculdade de Medicina da Universidade de São Paulo, São Paulo, Brazil; ^2^Laboratory of Neurosciences (LIM-27), Department and Institute of Psychiatry, Faculdade de Medicina da Universidade de São Paulo, São Paulo, Brazil

**Keywords:** bipolar depression, non-invasive brain electrical stimulation, clinical trial (RCT), blinding (masking), transcranial direct current electrical stimulation

We read the letter of Hu et al. (2018) commenting on our randomized clinical trial that examined the efficacy and safety of transcranial direct current stimulation (tDCS) in bipolar depression (Sampaio-Junior et al., 2018) with great interest. We believe that the authors have performed an adequate summary of our main study findings and limitations. Nonetheless, there are some issues that deserve further clarification.

First, the authors stated that the “*connections of the stimulator were concealed (…) [as to not] determine the polarity of stimulation*.” This is imprecise. We employed tDCS devices that automatically deliver active or sham stimulation according to a code that is inserted in the device's keypad, as done in our previous studies (Brunoni et al., 2013b, 2014, 2017; Valiengo et al., 2016). Therefore, there is no concealment of connections, nor blinding of the stimulation polarity.

Second, the authors suggested that sustained remission was not proven because “remission analysis” or “tDCS design” was not optimal. The most likely explanation for lack of statistically significant differences in remission is due to a low sample size and, hence, an underpowered analysis. We agree that a larger sample size would demonstrate more meaningful results. Nonetheless, the study design was a randomized clinical trial, which is considered the “gold standard” to prove causality associated with an intervention, and the remission analysis was based on cumulative (sustained) remission, a more robust and clinically meaningful outcome than remission at any given time point.

Third, the authors said that a “*guinea pig effect*” was caused “*as nearly three-fifths participants of each group identified the allocation group*.” It is unclear what the authors mean for “guinea pig effect,” as this term is not often used (and the authors provided no references for such term). From a sociology book (Brinkerhoff et al., 2007), such effect would occur “*when subjects' knowledge that they are participating in an experiment affects their response*” and would relate to social desirability, as subjects would behave as they think it would be expected by the examiners. According to this definition (the only one we were able to find), such effect occurs in *all* randomized clinical trials, regardless of intervention or blinding. Therefore, the author's association between a guinea pig effect and (supposedly) a lack of blinding is a *non-sequitur*. We highlight that the sham method used in our study was proven to be as reliable as the gold standard placebo-pill (Brunoni et al., 2013a). Although an active control (e.g., stimulation of another brain region) could be implemented in design, this would add additional difficulties in staff blinding who would identify the allocation group based on electrode positioning.

Fourth, the authors critically omitted that correct group guessing was not above chance. Importantly, although we indeed used group guessing as a proxy for blinding integrity, it is important to mention that correct guessing can occur due to (lack of) improvement. Such effect can be observed in Figure 1. In patients allocated to sham group, there was a statistically significant difference in terms of response (*p* < 0.001) between those who correctly guessed that they were in sham group (6.7%) and those who incorrectly guessed that they were in active group (63.6%). Likewise, in patients allocated to active group, there was a statistically significant difference in terms of response (*p* = 0.04) between those who incorrectly guessed they were in sham group (50%) and those who correctly guessed they were in active group (87.5%). Therefore, participants tended to guess they were in the active group if they presented response, and that they were in the sham group if they did not present response. Reverse causality is unlikely as, overall, patients in the active group responded twice as more than in sham group, guessing was not beyond chance, and tDCS blinding seems to be as effective as the gold-standard placebo pill (Brunoni et al., 2013a). For these reasons, routine blinding checking is not anymore recommended in randomized clinical trials (Schulz et al., 2010).

**Figure 1:**
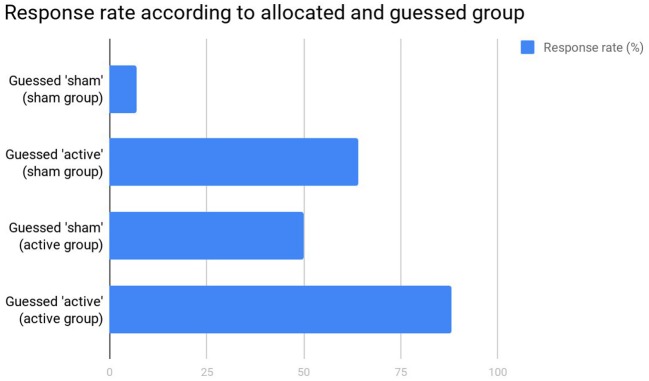
Response rates in the original study (Sampaio-Junior et al., 2018).

Fifth, the authors made some comments regarding the scales and randomization methods we adopted. It is important to underscore that the study methodology was published a priori (Pereira Junior Bde et al., 2015) and that it abides to the state-of-the-art methodology in clinical trial design. In hindsight, we agree that the Clinical Global Impression (CGI) was not the optimal choice for our sample and that other scales could have been used, such as the Bipolar Depression Rating Scale (Berk et al., 2007).

Finally, tDCS was well-tolerated, as only skin redness was statistically higher in the active vs. sham group. Moreover, although the rate of treatment-emergent affective switch (TEAS) was high, rates were similar in both groups. Importantly, TEAS was based on a Young Mania Rating Scale score >8. Clinically, these episodes did not meet the criteria for a major depressive episode with mixed features, hypomania, or mania and required no hospitalization, trial discontinuation, or specific treatment.

We agree that our trial presents limitations that demand further investigations of tDCS efficacy in bipolar depression. Considering the burden of disease, and the advantages of tDCS regarding portability and safety (Brunoni et al., 2018), showing that tDCS is effective for this condition would bring enormous clinical gains.

## Author Contributions

All authors listed have made a substantial, direct and intellectual contribution to the work, and approved it for publication.

### Conflict of Interest Statement

The authors declare that the research was conducted in the absence of any commercial or financial relationships that could be construed as a potential conflict of interest.
